# U-Omp19 from *Brucella abortus* Is a Useful Adjuvant for Vaccine Formulations against *Salmonella* Infection in Mice

**DOI:** 10.3389/fimmu.2017.00171

**Published:** 2017-02-17

**Authors:** Gabriela S. Risso, Marianela V. Carabajal, Laura A. Bruno, Andrés E. Ibañez, Lorena M. Coria, Karina A. Pasquevich, Seung-Joo Lee, Stephen J. McSorley, Gabriel Briones, Juliana Cassataro

**Affiliations:** ^1^Instituto de Investigaciones Biotecnológicas “Rodolfo Ugalde”-Instituto Tecnológico de Chascomús (IIB-INTECH), Universidad Nacional de San Martín (UNSAM), Consejo Nacional de Investigaciones Científicas y Técnicas (CONICET), Buenos Aires, Argentina; ^2^Center for Comparative Medicine (CCM), Department of Anatomy, Physiology and Cell Biology, School of Veterinary Medicine, University of California Davis, Davis, CA, USA

**Keywords:** adjuvant, vaccination, *Salmonella* Typhimurium, T helper 1, T helper 17

## Abstract

Most pathogens infect through mucosal surfaces, and parenteral immunization typically fails to induce effective immune responses at these sites. Development of oral-administered vaccines capable of inducing mucosal as well as systemic immunity while bypassing the issues of antigen degradation and immune tolerance could be crucial for the control of enteropathogens. This study demonstrates that U-Omp19, a bacterial protease inhibitor with immunostimulatory features, coadministered with *Salmonella* antigens by the oral route, enhances mucosal and systemic immune responses in mice. U-Omp19 was able to increase antigen-specific production of IFN-γ and IL-17 and mucosal (IgA) antibody response. Finally, oral vaccination with U-Omp19 plus *Salmonella* antigens conferred protection against virulent challenge with *Salmonella* Typhimurium, with a significant reduction in bacterial loads. These findings prove the efficacy of this novel adjuvant in the *Salmonella* infection model and support the potential of U-Omp19 as a suitable adjuvant in oral vaccine formulations against mucosal pathogens requiring T helper (Th)1–Th17 protective immune responses.

## Introduction

It is generally accepted that vaccination is the most efficient and cost-effective form of preventing infectious diseases. Although most vaccines currently licensed are administered by parenteral route, this vaccination strategy usually fails to elicit adequate mucosal immune responses. As a result, diseases caused by mucosal pathogens are still among the major causes of death in developing countries. It is well documented that oral immunization is capable of generating strong protective immunity at the intestinal mucosa as well as systemically (1–5). However, oral administration of antigens faces two major issues, one being degradation of the antigen by enzymes present at the gastrointestinal tract and the other being the induction of immune tolerance against the administered antigen (6–8). Current oral vaccines based on attenuated pathogens are usually capable of bypassing these difficulties but, on the other hand, present important safety concerns (9). The safe alternative consists on the development of oral killed or subunit vaccines, but this holds major challenges since they are poorly immunogenic and usually require multiple doses and the use of an effective oral adjuvant (10, 11).

Two bacterial products are being used as oral adjuvants in the mouse model, cholera toxin (CT) from *Vibrio cholerae* and heat-labile enterotoxin (LT) from *Escherichia coli* (12). Since enterotoxicity seriously limits the practical use of these compounds in humans, modifications have been generated to reduce this effect. A modified version of CT lacking the A subunit (CTB) is now currently licensed as part of the Dukoral^®^ vaccine for human use (13) and also a double mutant of LT, which retains its adjuvant properties is under clinical trial (14). However, neither of these molecules is capable of overcoming antigen degradation in the gastrointestinal tract. Interesting strategies to address this issue include antigen delivery through intestine-targeted pH-dependent microparticles (15), biodegradable nano or microparticles (16–18), and antigen targeting to M cells (19). However, there is still a need for an appropriate oral adjuvant, especially one that induces T helper (Th)1 and CD8^+^ T cell immune responses required for protective immunity against intracellular pathogens.

In previous work we have studied the unlipidated form of a bacterial protease inhibitor from *Brucella abortus*, U-Omp19, as an oral adjuvant. We have demonstrated that it has self-adjuvanting properties (20) and can also enhance immune responses against the model antigen chicken ovalbumin (OVA) (21, 22). The mechanisms responsible for the adjuvant activity of U-Omp19 rely in the inhibition of stomach and gut proteases and recruitment of immune cells to the gastrointestinal mucosa (21) as well as increased maturation of DCs and enhanced presentation of the antigen by means of delaying antigen digestion at the lysosomes (23). Thus, mucosal as well as systemic OVA-specific immune responses, Th1, and CD8^+^ are enhanced if U-Omp19 is codelivered orally.

We now focused on assessing U-Omp19’s capacity to increase immune response against antigens from an enteric pathogen that invades the host through the gastrointestinal mucosa. The enterobacteria *Salmonella* infect humans and animals and depending on the serotype can cause disease of different severity (24). *S*. Typhi invades the gut mucosa and is drained trough the lymphatic system into lymph nodes, spleen, and liver (25). The main structural protein of *Salmonella* flagellum is Flagellin (FliC). Flagellin was demonstrated to play an important role in the protection against *Salmonella* challenge (26). Also, a significant fraction of *Salmonella*-specific CD4^+^ T cells respond to FliC, and this antigen has the capacity to protect naive mice from lethal *Salmonella* infection (27). Another protein expressed and secreted by *Salmonella* is SseB, which promotes membrane pore formation, allowing proteins to access host cytoplasm. SseB was highlighted in a proteomic screen and has been shown to protect mice against *Salmonella* infection (28). Th1 immunity involving IFN-γ is strongly associated with the protective immune response to *Salmonella* (29–31). The natural route of infection and the need for a Th1-biased response, along with the current emergence of multidrug-resistant strains, makes this pathogen a strong candidate for oral vaccination with the novel adjuvant U-Omp19. The conventional mouse model for the study of this disease is infection of susceptible mouse strains with *Salmonella enterica* serovar Typhimurium (*S*. Typhimurium) that causes an invasive systemic disease that is similar in many respects to typhoid fever (30–32). Therefore, in this work U-Omp19 was studied *in vivo* for its ability to elicit mucosal and systemic immunity against coadministered antigens from *S*. Typhimurium. We assessed a killed-vaccine immunization alternative, using a heat-killed extract of *S*. Typhimurium (HKS) as well as a proof of concept using FliC and SseB antigens in a potential subunit vaccine alternative.

## Materials and Methods

### Ethics Statement

All experimental protocols of this study were conducted in agreement with international ethical standards for animal experimentation (Helsinki Declaration and its amendments, Amsterdam Protocol of welfare, and animal protection and National Institutes of Health, USA, guidelines: Guide for the Care and Use of Laboratory Animals). The protocols of this study were approved by the Institutional Committee for the Care and Use of Experimentation Animals from the University of San Martin (UNSAM) or from University of California Davis.

### Mice

BALB/c and CF-1 mice were obtained from Animal Facility at IIB-UNSAM or from Jackson Laboratory and were used at 6–12 weeks of age. Mice were housed in appropriate conventional animal care facilities and handled according to international guidelines required for animal experiments at IIB-UNSAM or at University of California Davis.

### Antigens and Adjuvants

The recombinant unlipidated (U-) Omp19 was expressed and purified as previously described (33). Lipopolysaccharide (LPS) contamination was adsorbed with Sepharose-polymyxin B (SIGMA, St. Louis, MO, USA). Endotoxin and protein concentration were determined as in Ref. (21). All U-Omp19 preparations used contained <0.10 endotoxin U/mg of protein. CT and CT subunit B (CTB) from *V. cholerae* were purchased from SIGMA, St. Louis, MO, USA.

For preparation of heat-killed extract of *Salmonella* Typhimurium (HKS), AroA *S*. Typhimurium (streptomycin resistant) was plated in LB agar with 10 µg/ml of streptomycin and grown overnight at 37°C. Afterward, eight isolated colonies were taken from the LB plate and grown in LB medium with 10 µg/ml of streptomycin at 37°C with agitation of 130 rpm overnight. The next morning, the culture was diluted 1 in 25 in LB medium with 10 µg/ml of streptomycin and grown until reaching an OD 600 nm of 0.85 (≈1 × 10^9^ CFU/ml), for the bacterial culture to be in growth phase. After centrifugation for 10 min at 6,000 rpm at 4°C, supernatant was discarded and bacterial pellet was suspended in sterile PBS. This bacterial extract was then heated at 63°C for 3 h; afterward it was heated at 100°C for 10 min and finally sonicated for 10 min in an ice-cold environment. Protein concentration was determined by the bicinchroninic acid assay (Pierce, Rockford, IL, USA) and aliquots of 1–5 × 10^6^ CFU/μg of extract were stored at −70°C until used.

SseB was obtained as described in Ref. (28). Flagellin was purified from LPS-deficient *S*. Typhimurium X4700 as in Ref. (34).

### Antigen Degradation Studies

To obtain the mouse stomach extract, five mice were fasted overnight. Stomach was obtained from every mouse and mechanically homogenized in 500 µl of ice-cold PBS (Ultra-Turrax homogenizer, IKA). Homogenates were pooled, immediately centrifuged 15 min at 6,000 rpm, and supernatants were used as mouse stomach extract. Standardization of extracts was done by measuring (i) total protein content by the bicinchroninic acid assay (Pierce, Rockford, IL, USA) and (ii) protease activity of the extract. Protease activity was determined using a casein fluorimetric kit (EnzChek, Invitrogen, Carlsbad, CA, USA). EnzChek kit contains casein BODIPY FL, a protein in which fluorescence is quenched. Protease-catalyzed hydrolysis releases this quenching, yielding bright green fluorescent peptides. The increase in fluorescence is proportional to protease activity.

To examine whether U-Omp19 would limit digestion of HKS by stomach proteases, HKS was incubated with stomach extracts with or without U-Omp19 for 5 h. Following incubation, each mixture of reaction was separated on 12% SDS-PAGE. To increase sensitivity of this assay we performed Western Blot against FliC (*Salmonella* Flagellum Protein-FliC-) an antigen that has shown to be protective in mice (26, 27). Membrane was incubated overnight with anti-FliC IgG MAb (Invivogen Carlsbad, CA, USA), followed by 1-h incubation with anti-IgG-HRP (SIGMA, St. Louis, MO, USA), and reveled using ECL kit (Pierce, Rockford, IL, USA). This helped us determine if U-Omp19 can protect digestion of this particular protein in the heat-killed preparation of *Salmonella*.

### Immunization

BALB/c and CF-1 mice were immunized orally—intragastrically by oral gavage—with HKS (100 µg ≈ 1–5 × 10^8^ CFU) alone, with U-Omp19 (200 µg), with CTB or with CT (10 µg) on days 0 and 7. These were diluted in PBS until a final volume of 200 µl per administration per mouse. Dose of U-Omp19 was chosen considering previous studies on protease inhibitory capacity of U-Omp19 *in vivo* (21), whereas CT dose was selected for being the typical effective oral dose described in the literature (35–38). Mice were fasted 2 h before and after immunization. Fifteen minutes before oral immunization, mice were administered with 100 µl of 0.1M sodium bicarbonate.

For subunit vaccine experiments, BALB/c mice were intravenously or orally immunized on days 0 and 30 with (i) buffer, (ii) 20 µg SseB + 20 µg Flagellin + 80 µg U-Omp19, or (iii) 20 µg SseB + 20 µg Flagellin + 10 µg monophosphoryl lipid A (MPLA) (Invivogen, Carlsbad, CA, USA) as a control adjuvant.

### Determination of Th Immune Responses

Spleen and MLNs’ single cell suspensions from immunized mice were obtained as in Ref. (21) 14 days post-last immunization and cells’ suspensions were cultured in the presence of 0.5 or 10 micrograms/ml of HKS or complete medium. After 3 days, cell culture supernatants were collected and IFN-γ, IL-4, IL-10, and IL-17 were determined by ELISA (Pharmingen, San Diego, CA, USA).

### Delayed Type Hypersensitivity Assay (DTH) Test

Two weeks after the last immunization, mice were injected intradermally in one footpad with 20 µg of HKS in 40 µl of saline and in the contralateral footpad with an equal volume of saline. Footpad thickness was measured 72 h later using a digital caliper with a precision of 0.01 mm, and the mean increase in footpad thickness (mm) was calculated as (footpad thickness) HKS − (footpad thickness) saline.

### Determination of IgA in Feces and IgG in Sera

Two weeks after the last immunization, fecal extracts were prepared as in Ref. (20) and used on the same day. Mice were bled *via* the cheek pouch on the submandibular vein. Blood was allowed to clot, and serum was removed and stored at −20°C until used.

For ELISA, HKS extract was diluted to 1 mg/ml in NaHCO_3_ 1M pH 9.6, and ELISA plates were coated with 100 µg/well overnight at 4°C. Plates were blocked with bovine serum albumin 2% in PBS at 37°C for 1 h and washed with PBS-Tween 0.05%. Samples were incubated for 2 h at room temperature and after washing, anti-mouse IgA-HRP (Abcam, Cambridge, MA, USA) or anti-mouse IgG-HRP (SIGMA, St. Louis, MO, USA) diluted in PBS–BSA 1% were added for 1 h at room temperature. Finally, detection was performed with BD OptEIA™ TMB Substrate Reagent Set (BD, San Diego, CA, USA). Anti-U-Omp19 ELISA was performed as previously described (33).

### Protection Experiments

#### HKS Experiments

For BALB/c mice, 14 days post-last immunization, mice from each group were challenged intragastrically with 0.5–1 × 10^5^ CFU of virulent *S*. Typhimurium. For CF-1 mice, 21 days post-last immunization, mice from each group were challenged intragastrically with 1–5 × 10^5^ CFU of virulent *S*. Typhimurium. Mice were fasted for 6 h before and 1 h after infection. Fifteen minutes before infection mice were administered intragastrically with 100 µl of NaHCO_3_ 0.1M pH8. Sacrifice was performed 6 days postinfection for BALB/c mice and 40 days postinfection for CF-1 mice. Spleens and livers were obtained and homogenized in sterile PBS. Serial dilutions of the homogenates were plated on SS agar plates and incubated overnight at 37°C to determine bacterial colonization.

#### Recombinant Antigens Experiments

Thirty days post-last immunization, mice were intravenously (tail vein) infected with 1,000 CFU of virulent *Salmonella* SL1344 strain. Four days later spleen and liver were obtained, and bacterial load was determined by homogenization, serial dilution, and plating on MacConkey agar plates.

### Statistical Analysis

Statistical analysis and plotting were performed using GraphPad Prism 5 software. Data (with logarithmic transformation when necessary) were tested for normality and homoscedasticity before using parametric statistics (one-way ANOVA) or analyzed using non-parametric statistics (Kruskal–Wallis). Data were tested for normality using the Kolmogorov–Smirnov test, and for equal variance, using the Levene Median test. Results shown are representative of at least two independent experiments. Results were expressed as mean ± SEM. Significance level was set at *p* < 0.05.

## Results

### U-Omp19 Protects HKS Antigens from Stomach Digestion

Previous studies have shown that U-Omp19 has inhibitory activity over several proteases at the digestive tract, particularly pepsin, the main protease present at the stomach (21). Thus, U-Omp19’s activity in an *in vitro* degradation assay incubating HKS with mouse stomach extract was evaluated. One of the antigens present in the HKS extract is FliC. FliC is a major protein and virulence factor from *Salmonella* that has the capacity to protect naive mice from lethal *Salmonella* infection (26–28), so if degradation of FliC by stomach enzymes is reduced, more FliC can reach inductive sites and thus it is likely to be able to perform as a protective antigen in an oral vaccine. Detection of FliC by western blot confirms that U-Omp19 protects antigen from degradation by stomach enzymes *in vitro* (Figure S1 in Supplementary Material), which may contribute to the induction of a stronger immune response.

### Vaccination with HKS Plus U-Omp19 Induces an Increase in Mucosal Antibody Response

**Figure 1:**
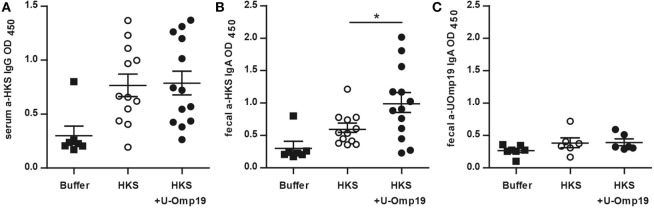
**U-Omp19 induces an increase in fecal IgA antibodies against HKS**. BALB/c mice were orally immunized on days 0 and 7 with buffer, HKS alone, or HKS + U-Omp19. Fourteen days post-last immunization, sera and feces were collected. HKS-specific IgG in serum **(A)**, HKS-specific IgA in fecal extracts **(B)**, and U-Omp19-specific IgA in fecal extracts **(C)** were detected by ELISA. Individual mice are shown as scatter dots (*p < 0.05; ANOVA plus Bonferroni’s multiple comparisons test).

There is evidence that antibodies are associated with protection against *S*. Typhimurium (39, 40). To assess U-Omp19’s adjuvant effect on the antibody response against HKS antigens, HKS-specific antibody production in orally immunized mice was examined. Intestinal HKS-specific IgA antibodies were significantly elevated in fecal pellets from mice immunized with HKS plus U-Omp19 compared to those immunized with HKS alone. Serum HKS-specific IgG levels, on the other hand, were not altered if U-Omp19 was coadministered with HKS. Of note, immunization with U-Omp19 as adjuvant did not induce a mucosal antibody response against itself (Figure 1).

### U-Omp19 Induces Systemic and Mucosal Cellular Immune Responses against Coadministered HKS Antigens

**Figure 2:**
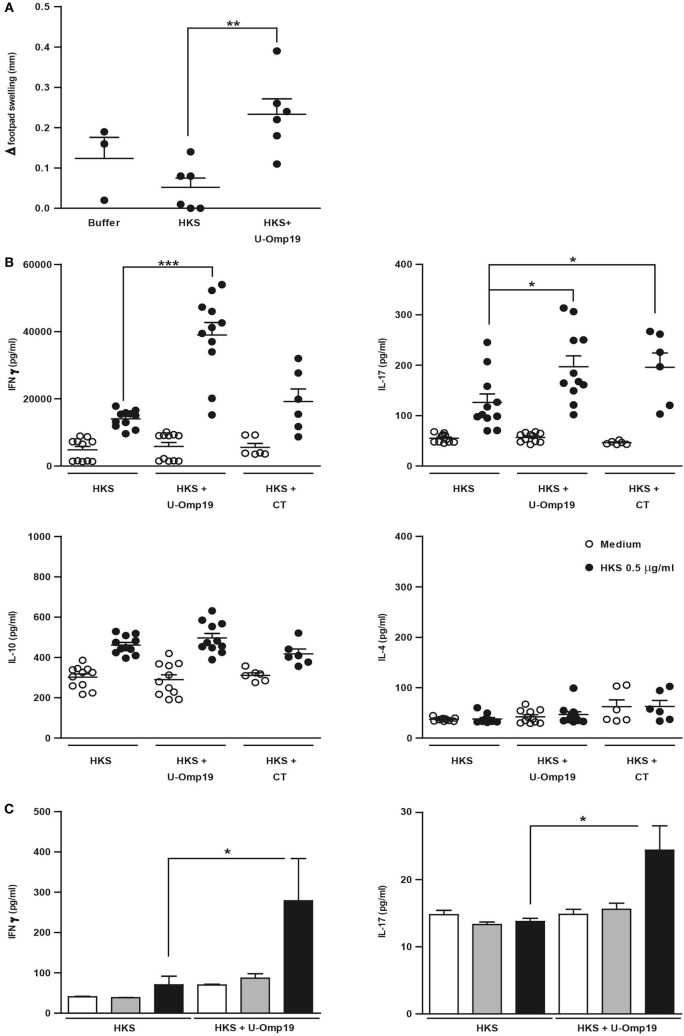
**U-Omp19 increases systemic and mucosal cellular immune response against HKS**. **(A)** BALB/c mice were orally immunized on days 0 and 7 with buffer, HKS alone, or HKS + U-Omp19. Fourteen days post-last immunization, mice received an intradermal injection of HKS on one footpad and saline on the contralateral footpad. Swelling of each footpad was measured with a caliper 72 h later, and the difference between saline and HKS injected footpad was analyzed (**p < 0.01; ANOVA plus Bonferroni’s multiple comparisons test). **(B)** BALB/c mice were orally immunized on days 0 and 7 with HKS alone, HKS + U-Omp19, or HKS + CT. Spleens were obtained 14 days post-last immunization, and cells’ suspensions were cultured in the presence of 0.5 μg/ml of HKS or complete medium. Supernatants were collected after 3 days, and cytokine production was determined by ELISA. Results from individual mice are shown as scatter dots (*p < 0.05, ***p < 0.001 vs HKS; Kruskall–Wallis plus Dunn’s multiple comparisons test). **(C)** BALB/c mice were orally immunized on days 0 and 7 with HKS alone or HKS + U-Omp19. MLNs were obtained 14 days post-last immunization and, cells’ suspensions were cultured in the presence of 0.5 or 10 µg/ml of HKS or complete medium. Supernatants were collected after 3 days, and cytokine production was determined by ELISA (*p < 0.05; Kruskal–Wallis plus Dunn’s multiple comparisons test).

Since the cellular immune response has been reported to be important for protection against *Salmonella* (41–43), the capacity of U-Omp19 to increase HKS-specific cell-mediated immune response in a DTH assay was tested in immunized mice. After 72 h of intradermal administration of HKS, mice that had been previously delivered with HKS + U-Omp19 displayed an increased DTH response compared to the HKS-immunized group (Figure 2A). The profile of cytokine production upon antigen stimulation was studied after intragastric immunization with HKS, HKS + U-Omp19, or HKS + CT as mucosal adjuvant control. Splenocytes from HKS + U-Omp19 immunized mice presented a significant increase in IFN-γ and IL-17 production upon HKS stimulation *in vitro* in comparison to HKS alone immunization. In contrast, HKS stimulation *in vitro* did not increase IL-4 or IL-10 production (Figure 2B). CT induced a statistically significant increase in IL-17 and a slight but not significant increase in IFN-γ.

To evaluate U-Omp19’s effect on HKS-specific mucosal immune response, MLNs from immunized mice were obtained and stimulated *in vitro* with HKS. As seen in splenocytes, IFN-γ and IL-17 levels in supernatants from MLNs’ cells were increased in animals immunized with HKS + U-Omp19 compared to HKS alone (Figure 2C). Production of IL-4 and IL-10 did not differ between groups (data not shown).

### Coadministration of HKS with U-Omp19 Reduces Bacterial Burden after Oral Challenge with *S*. Typhimurium

**Figure 3:**
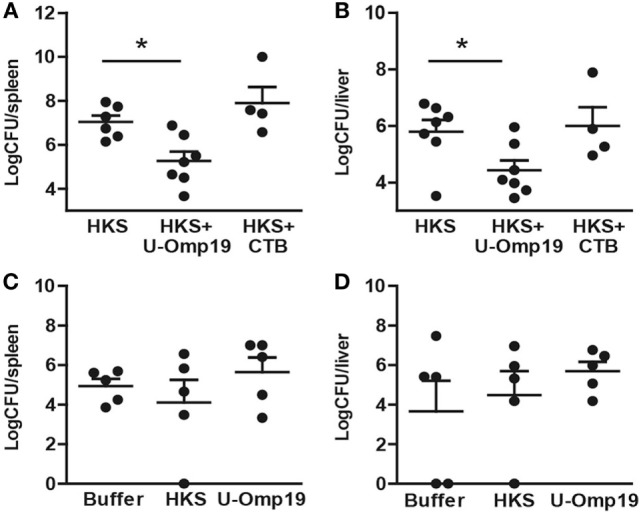
**Immunization with U-Omp19 + HKS reduces bacterial burden after *Salmonella* infection**. **(A,B)** BALB/c mice were orally immunized on days 0 and 7 with HKS alone, HKS + U-Omp19, or HKS + CTB. Fourteen days post-last immunization, mice were orally infected with *Salmonella* Typhimurium. Six days postinfection mice were sacrificed, spleen **(A)** and liver **(B)** were harvested, and homogenates were plated on SS agar plates. **(C,D)** BALB/c mice were orally immunized on days 0 and 7 with HKS alone, buffer, or U-Omp19 alone. Fourteen days post-last immunization, mice were orally infected with *S*. Typhimurium. Six days postinfection mice were sacrificed, spleen **(C)** and liver **(D)** were harvested, and homogenates were plated on SS agar plates. CFU were counted after overnight incubation at 37°C. Individual mice are shown as scatter dots (*p < 0.05 vs HKS; ANOVA plus Bonferroni’s multiple comparisons test).

To evaluate if the induction of an adaptive immune response by HKS + U-Omp19 would lead to an increased level of protection against infection, immunized mice were orally infected with *S*. Typhimurium. Animals that received HKS + U-Omp19 presented a significant reduction in bacterial burden in spleen and liver compared to those that were administered with HKS alone (Figures 3A,B), indicating that addition of U-Omp19 to the HKS extract can increase protection against oral *S*. Typhimurium challenge. In contrast, animals immunized with HKS + CTB were not protected against infection. Importantly, U-Omp19 orally delivered alone did not induce reduction in bacterial burden when compared with buffer or HKS delivered mice (Figures 3C,D).

### U-Omp19 Maintains Its Adjuvant Capacity in a Chronic Outbred Mouse Model

**Figure 4:**
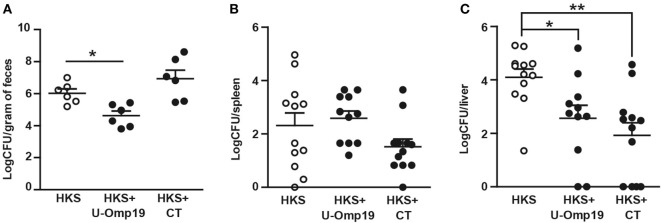
**Coadministration of U-Omp19 with HKS effectively reduces bacterial colonization in outbred mice after infection with *Salmonella***. CF-1 mice were orally immunized on days 0 and 7 with HKS alone, HKS + U-Omp19, or HKS + CT. Twenty-one days post-last immunization, mice were orally infected with *Salmonella* Typhimurium. **(A)** Four weeks postinfection, fecal pellets were suspended in PBS and plated on SS agar plates. For organ colonization experiments, 40 days postinfection mice were sacrificed, spleen **(B)** and liver **(C)** were harvested, and homogenates were plated on SS agar plates. CFU were counted after overnight incubation at 37°C. Individual mice are shown as scatter dots (*p < 0.05, **p < 0.01; Kruskal–Wallis plus Dunn’s multiple comparisons test).

Though outbred animals may cause more variability in the experiments, they are more akin to the human population. Therefore, U-Omp19’s adjuvant capacity in the CF-1 outbred mice strain was evaluated. Unlike BALB/c mice, CF-1 mice present a functional Nramp1 gene, they are notably less susceptible to *Salmonella* infection, and develop a chronic disease (30), which allows protection studies to be performed at longer time periods. Coadministration of U-Omp19 was able to enhance the protective capacity of HKS in this mouse strain, evidenced by a reduction in fecal shedding of bacteria and significantly lower CFU counts in liver compared to HKS alone. Immunization with HKS + CT had no effect on fecal shedding but produced a significant reduction in bacterial burden at the liver (Figure 4).

### U-Omp19 When Coadministered with Recombinant *Salmonella* Proteins Reduces Bacterial Burden after *Salmonella* Infection

**Figure 5:**
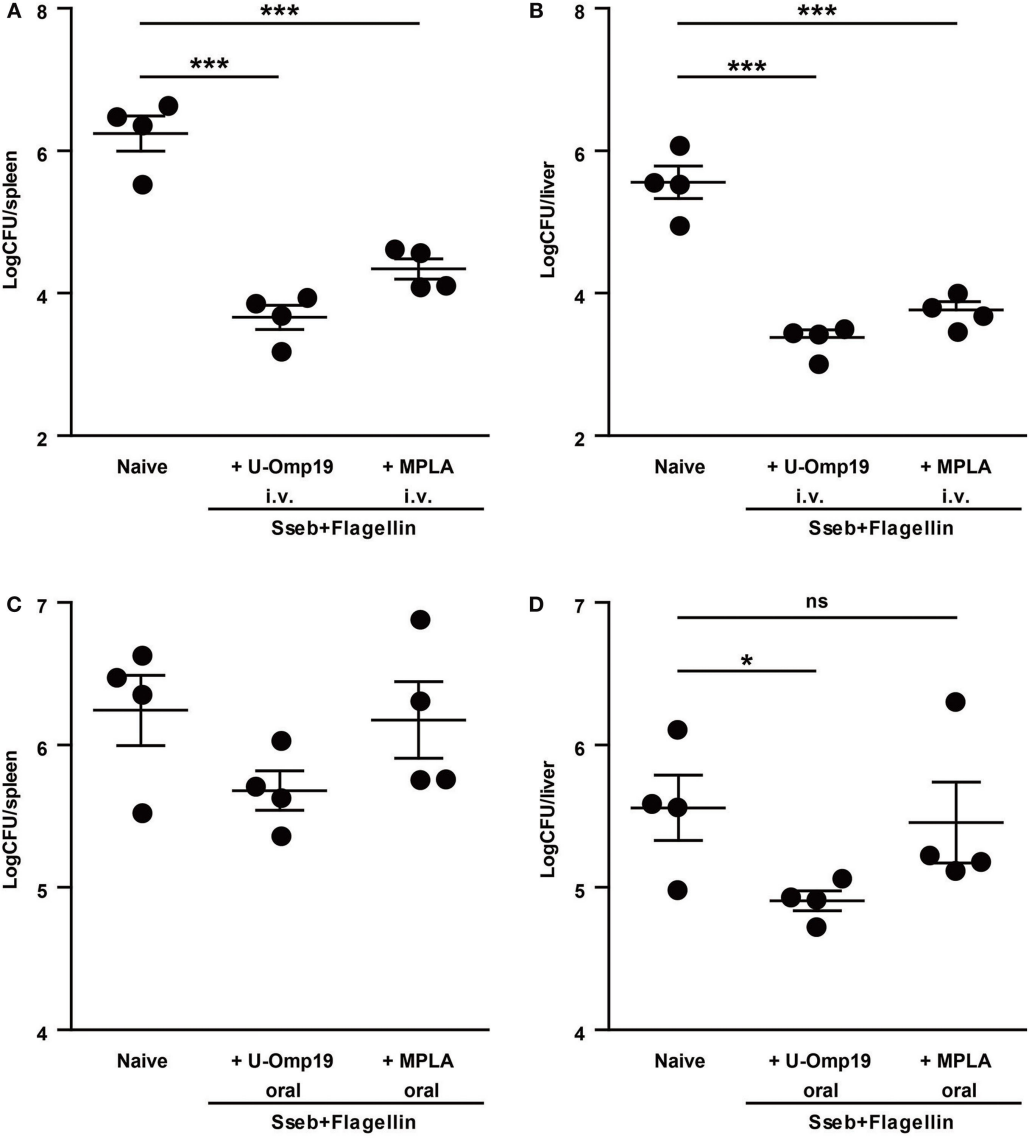
**U-Omp19 coadministered with recombinant *Salmonella* proteins reduces bacterial burden after *Salmonella* Typhimurium infection**. BALB/c mice were immunized i.v. **(A,B)** or orally **(C,D)** on days 0 and 30 with buffer, SseB + Flagellin + U-Omp19, or SseB + Flagellin + MPLA. Thirty days post-last immunization, mice were i.v. (tail vein) infected with virulent *S*. Typhimurium. Four days later, spleen **(A,C)** and liver **(B,D)** were obtained, and bacterial load was determined by homogenization, serial dilution, and plating on MacConkey agar plates (*p < 0.05, ***p < 0.001; ANOVA plus Bonferroni’s multiple comparisons test).

Subunit vaccination is an extremely safe method of immunization, it can be used on virtually everyone in need of vaccination regardless of health status and for that reason we sought to assess U-Omp19’s adjuvant capability using recombinant *Salmonella* antigens. We chose two antigens that have previously been reported to reduce CFU count after infection: SseB, a part of the *Salmonella* Pathogenicity Island II, and Flagellin, which is the major structural protein of *Salmonella* flagellum (26–28). Preliminary studies showed that combination of these two antigens increases protective efficacy (S. J. McSorley, personal communication). Since U-Omp19 proved to have adjuvant properties when coadministered with recombinant protein antigens orally as well as systemic (21, 22), both routes were evaluated. In the systemic (i.v.) immunization protocol, U-Omp19 was as efficient as control adjuvant MPLA in reducing CFU counts after infection at spleen and liver of immunized mice compared to naive mice. Of note, in the oral immunization protocol, MPLA adjuvant was unable to induce protection against bacterial challenge, whereas mice immunized with U-Omp19 presented significantly lower bacterial burden at the liver (Figure 5).

## Discussion

Although most vaccines currently licensed are administered by systemic route, parenteral vaccination strategies fail to generate an adequate local mucosal immune response against still prevailing intestinal infections. On the contrary, oral immunization is capable of generating strong protective immunity at the intestinal mucosa as well as systemic (1–5). Particularly in developing countries, oral vaccination may offer a means to deal with safety concerns (associated with needle use) and the need for mass vaccination. However, in order to elicit an appropriate immune response, antigen digestion by proteases at the gastrointestinal tract must be avoided. Previous studies have shown that U-Omp19 has inhibitory activity over several proteases, in particular Pepsin present at the stomach (21). These results explain the observed protection of the HKS representative antigen flagellin from proteases present in a mouse stomach extract. As proteases at the stomach are constantly renewed, the effect of U-Omp19 consists in a delay of the HKS extract degradation increasing its half-life. Using chicken OVA as a model antigen, it was demonstrated that this delay in antigen degradation increases the amount of antigen that reaches mucosal inductive sites (21). This may also be the case in the HKS model contributing to the induction of a stronger immune response against this antigen. Of note, study of other protease inhibitors has shown that the protease inhibitory activity by itself does not induce immune response against the coadministered antigen; it is the combination of U-Omp19’s immunostimulatory properties together with a delay in antigen degradation that explain the adjuvant properties of U-Omp19 (21, 23).

The role of IgA in resistance against *Salmonella* infection is still controversial. Some state that antibodies against LPS, one of the components in the HKS extract, are associated to protection by blocking adhesion of bacteria to epithelial cells (39, 40), others declare that IgA is not required for protection since IgA-deficient mice can be fully protected from infection (28), while others propose that the primary role of B cells in acquired immunity to *Salmonella* is *via* the development of protective T cell immunity (44), so the IgA increment would not be important *per se* but merely an immune correlate of B cell activation. In this work we demonstrated that after one oral boost, U-Omp19 can increase the amount of HKS-specific IgA in feces. Therefore, a role of this antibody increment in increased protection could be plausible. Also, coadministration of U-Omp19 did not induce a mucosal antibody response against itself, meaning that there would be no reduction of the adjuvant effect in a potential subsequent oral administration.

Cellular immune response has also been reported to be important for protection against *Salmonella* (41). Th1 cytokines and IFN-γ in particular seem to be crucial during the initial stages of *Salmonella* growth since IFN-γ^−/−^ mice develop high bacterial burden and succumb rapidly to infection (42). IL-17 as well has been implicated in the suppression of intestinal invasion by *Salmonella* (43). In previous work we described the role of U-Omp19 as self-adjuvant and adjuvant of model antigens (OVA) capable of shifting the immune response toward a Th1–Th17 profile (20, 21). In this work we provide additional evidence that supports the role of U-Omp19 as a Th1–Th17 oral adjuvant using antigens from a real pathogen. U-Omp19 was capable of inducing cell-mediated immune response in a DTH assay while increasing IFN-γ and IL-17 production in splenocytes from immunized mice, which is consistent with a strong induction of a systemic immune response upon oral immunization with U-Omp19 as adjuvant. In these experiments, CT was able to induce an increase in IL-17 production, as previously reported in cell culture and mucosal immunization models for this adjuvant (45–47).

The mucosa is the front line of host defense against most pathogens. Therefore, induction of local immune response is essential to enhance the mucosal immune barrier that prevents infection by enteric pathogens (48). Our findings reveal that U-Omp19 is capable of inducing increased levels of IFN-γ and IL-17 at the MLNs, indicating the triggering of local immune response. These results are in accordance with previous findings using OVA as model antigen (21).

Moreover, the overall effect of coadministration of U-Omp19 with the antigens from HKS on antibody and cellular immune responses resulted in an increase in protection against challenge with a lethal dose of virulent *S*. Typhimurium in both inbred BALB/c mice and outbred CF-1 mice. This proves that U-Omp19 is effective in different genetic backgrounds despite their intrinsic variability. Since U-Omp19 had no effect *per se* on protection against infection, the protective efficacy of the vaccine can only be attributed to U-Omp19’s adjuvant effect when coadministered with the antigen. Overall the adjuvant capacity of U-Omp19 on inducing protection against *Salmonella* infection was superior to CTB in BALB/c and similar to CT in CF-1 mice. As CT cannot be administered orally to humans because of its enterotoxic effects, our results open new options in the progress of oral-killed vaccine formulations against *Salmonella*.

In addition to circumventing safety concerns, subunit vaccines present further advantages to inactivated vaccines: since their exact composition is known, they can be more reproducible, and also there is no need to manipulate the virulent microorganism during vaccine manufacture. For sure the future of vaccinology is headed toward subunit vaccine production. In this work we were able to demonstrate that U-Omp19 is a suitable adjuvant in subunit vaccine formulations. Coadministration of U-Omp19 with recombinant *Salmonella* antigens intravenously was capable of reducing bacterial burden at spleen and liver after infection. Remarkably, U-Omp19 worked slightly better than MPLA, a well-known adjuvant approved by the U.S. Food and Drug Administration and used since 2009 in the Human Papillomavirus vaccine, Cervarix combined with aluminum hydroxide by the intramuscular route (49). Although i.v. immunization route is not applicable to human vaccination, our results show that this combination of antigens and U-Omp19 is capable of inducing protection against *Salmonella* infection by the i.v. route and encourages new studies combining these antigens and adjuvant with others and involving different means of vaccine delivery of relevance to human vaccination in the future.

Moreover, MPLA coadministered with the antigens by the oral route showed no signs of protection against infection, whereas U-Omp19 was able to induce a protective immune response evidenced by a significant reduction in bacterial burden at the liver of infected mice. These results open up a new range of possibilities for the rational design of oral subunit vaccines combining different recombinant antigens together with U-Omp19.

Full protection against infection is hard to achieve in this experimental model. However, the overall effect of coadministration of U-Omp19 with the antigens from HKS on antibody and cellular immune responses resulted in reduction of CFU counts after challenge with a lethal dose of virulent *S*. Typhimurium in both inbred BALB/c mice and outbred CF-1 mice. In the field of oral subunit vaccines, full protection is rarely achieved (30), and therefore, the reduction in bacterial load due to U-Omp19’s adjuvant effect is a very encouraging scenario and calls for more studies to continue to optimize protective efficacy. Oral vaccine formulations containing newly discovered antigens and U-Omp19 potentially combined with other delivery systems and adjuvants, as is the case in many combined-adjuvant vaccines, could fully prevent *Salmonella* infection.

In conclusion, these findings indicate that oral coadministration of HKS with U-Omp19 can enhance both systemic and mucosal immunity against HKS and shift the immune response toward a Th1/Th17-like profile, and this effect translates into an increased level of protection against infection with *S*. Typhimurium.

## Author Contributions

GR and JC designed the experiments. GR lead all laboratory assays with assistance from MC, LB, AI, and LC. S-JL and LB performed subunit vaccine experiment. GR, JC, and KP performed all statistical analysis. SM and GB provided bacterial strains and, together with JC, contributed with their expertise on the subject. GR and JC interpreted all results and wrote the manuscript. All authors reviewed, commented, and approved the manuscript.

## Conflict of Interest Statement

The authors declare that the research was conducted in the absence of any commercial or financial relationships that could be construed as a potential conflict of interest. LC, AI, KP, and JC are inventors on a patent related to U-Omp19. This patent, presented by the authors’ National Research Council, “Adjuvant for vaccines, vaccines that comprise it and uses,” presentation P 20090104015, was filed on October 19, 2009 in the National Institute of Intellectual Property, Argentina. This patent was also filed on October 18, 2010 in the European Patent Office, Spain PCT/ES2010/070667. EP2491946B1, CN102711815B, and DK2491946T3 granted. The filing of the patent did not have any role in experimental design, data collection and analysis, decision to publish, or preparation of this manuscript.
