# Supplementary figures and images for: U-Omp19 from Brucella abortus Is a Useful Adjuvant for Vaccine Formulations against Salmonella Infection in Mice
Source: Front Immunol. 2017 Feb 17;8:171. doi: 10.3389/fimmu.2017.00171 (PMC5313482; doi:10.3389/fimmu.2017.00171)

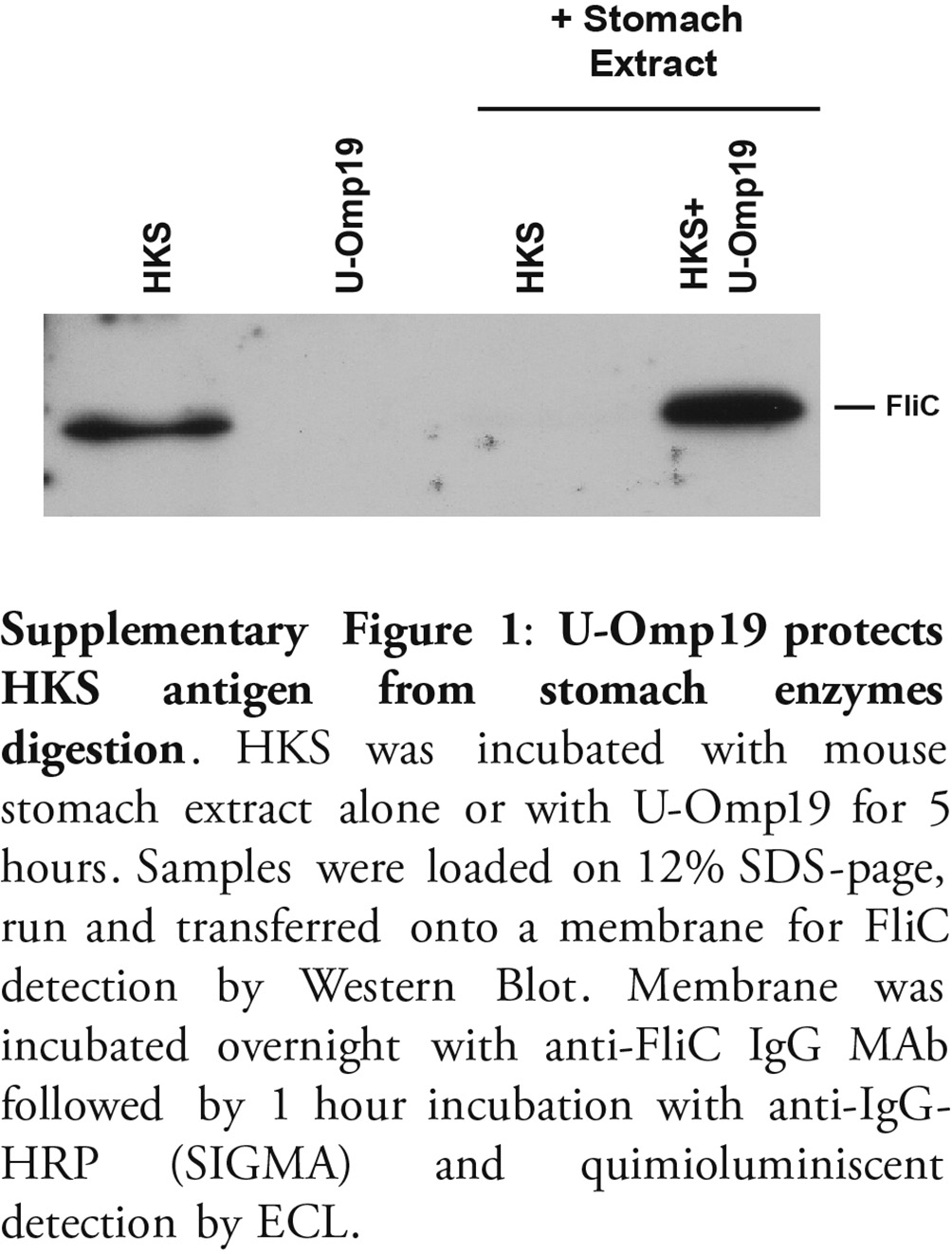

Supplement: Supplementary file 1 [file Image_1.JPG]
